# Cytokine‐Driven Hyperinflammation in Long COVID: Mechanisms, Biomarkers, Complement Dysregulation, and Emerging Immunotherapies—A Narrative Review

**DOI:** 10.1002/hsr2.73082

**Published:** 2026-08-26

**Authors:** Emmanuel Ifeanyi Obeagu

**Affiliations:** ^1^ Division of Haematology, Department of Biomedical and Laboratory Science Africa University Mutare Zimbabwe; ^2^ Department of Molecular Medicine and Haematology, Faculty of Health Sciences University of the Witwatersrand Johannesburg South Africa; ^3^ Department of Hematology and Immunohematology Bahir Dar University Bahir Dar Ethiopia

**Keywords:** cytokine storm, hyperinflammation, immunomodulation, long COVID, therapeutic strategies

## Abstract

**Background and Aims:**

Long COVID, also known as post‐acute sequelae of SARS‐CoV‐2 infection (PASC), is a multisystem condition characterized by persistent symptoms that continue beyond the acute phase of infection. Growing evidence indicates that sustained immune dysregulation involving cytokine‐mediated inflammation, complement activation, endothelial dysfunction, thromboinflammation, and viral antigen persistence contributes to its pathogenesis. This narrative review summarizes current evidence on the mechanisms underlying hyperinflammation in Long COVID, highlights emerging biomarkers, and evaluates evolving immunotherapeutic strategies.

**Methods:**

A narrative literature search was conducted using PubMed, Scopus, Web of Science, Embase, and Google Scholar for studies published between 2020 and 2026. Priority was given to systematic reviews, meta‐analyses, cohort studies, mechanistic investigations, and clinical trials examining immune dysregulation, inflammatory biomarkers, complement pathways, and targeted therapies in long COVID.

**Results:**

Persistent elevation of pro‐inflammatory cytokines, including interleukin‐6, interleukin‐1β, tumor necrosis factor‐α, interferon‐γ, and interleukin‐17, together with activation of the alternative and lectin complement pathways, contributes to endothelial injury, microvascular thrombosis, neuroinflammation, fibrosis, and multisystem dysfunction. Emerging biomarkers include inflammatory cytokines, complement proteins, endothelial activation markers, coagulation indices, and multi‐omics signatures that may improve disease stratification and therapeutic monitoring. Investigational therapies include cytokine‐targeted biologics, complement inhibitors, Janus kinase inhibitors, mesenchymal stem cell therapy, microbiome‐directed interventions, and precision immunotherapy.

**Conclusion:**

Long COVID results from complex interactions between persistent inflammation, complement dysregulation, vascular injury, and immune dysfunction. Integrating validated biomarkers with precision immunotherapeutic approaches may improve diagnosis, risk stratification, and individualized management. However, robust prospective studies and randomized clinical trials remain essential to validate these strategies and optimize long‐term clinical outcomes.

**Abbreviations**
ACE: angiotensin‐converting enzyme
B‐cell: B lymphocyte
CCL2: C‐C motif chemokine ligand 2
CNS: central nervous system
COVID‐19: coronavirus disease 2019
CRP: C‐reactive protein
CXCL10: C‐X‐C motif chemokine ligand 10
IFN‐γ: interferon gamma
IL‐1β: interleukin 1 beta
IL‐6: interleukin 6
IVIG: intravenous immunoglobulin
JAK: janus kinase
NET: neutrophil extracellular trap
NLRP3: NOD‐, LRR‐ and pyrin domain‐containing protein 3
PASC: post‐acute sequelae of SARS‐CoV‐2 infection (Chronic COVID)
PD‐1: programmed cell death protein 1
SARS‐CoV‐2: severe acute respiratory syndrome coronavirus 2
STAT: signal transducer and activator of transcription
TIM‐3: T‐cell Immunoglobulin and mucin‐domain containing‐3
TLR: toll‐like receptor
TNF‐α: tumor necrosis factor alpha
T‐cell: T lymphocyte
↑: elevated
↑↑: markedly elevated

## 1 Introduction

Long COVID, also referred to as post‐acute sequelae of SARS‐CoV‐2 infection (PASC), is a heterogeneous multisystem disorder characterized by persistent or newly emerging symptoms that continue for at least 3 months after acute SARS‐CoV‐2 infection and cannot be explained by an alternative diagnosis. It affects individuals across all age groups and disease severities, including those who experienced mild or asymptomatic acute infection. Common manifestations include fatigue, post‐exertional symptom exacerbation, cognitive dysfunction (“brain fog”), dyspnea, chest pain, palpitations, autonomic dysfunction, sleep disturbances, musculoskeletal pain, gastrointestinal symptoms, and neuropsychiatric complications, substantially impairing quality of life and functional capacity. Current estimates suggest that approximately 10%–30% of individuals infected with SARS‐CoV‐2 develop persistent symptoms, making Long COVID a major public health challenge with considerable socioeconomic implications [1, 2, 3]. The biological mechanisms underlying Long COVID are multifactorial and remain incompletely understood. Increasing evidence suggests that persistent viral antigen reservoirs, immune dysregulation, autoimmunity, endothelial dysfunction, thromboinflammation, mitochondrial impairment, dysbiosis of the gut microbiome, and reactivation of latent viruses collectively contribute to disease persistence. Rather than being driven by a single pathogenic pathway, long COVID appears to result from the interaction of several overlapping inflammatory and immunological processes that vary among individuals and clinical phenotypes [4, 5].

Persistent immune activation is one of the most consistently reported features of Long COVID. Sustained elevations of proinflammatory cytokines—including interleukin (IL)‐6, IL‐1β, tumor necrosis factor‐alpha (TNF‐α), interferon‐gamma (IFN‐γ), IL‐17, and multiple chemokines—have been associated with chronic fatigue, neurocognitive impairment, pulmonary dysfunction, cardiovascular abnormalities, and ongoing tissue injury. These cytokines promote chronic activation of innate and adaptive immune cells, amplify inflammatory signaling, disrupt endothelial homeostasis, and contribute to fibrosis and impaired tissue repair. However, cytokine dysregulation alone does not fully explain the complex pathophysiology of Long COVID [6, 7]. Recent landmark studies have identified persistent complement dysregulation as another major driver of Long COVID pathogenesis. Longitudinal proteomic analyses have demonstrated sustained activation of the alternative complement pathway, accompanied by persistent thromboinflammation, endothelial injury, platelet activation, and impaired fibrinolysis months after acute infection [8]. Similarly, Baille et al. [9] reported that complement dysregulation is highly prevalent in Long COVID and represents a potentially treatable biological pathway. In addition to the alternative pathway, increasing evidence indicates that persistent SARS‐CoV‐2 spike and nucleocapsid proteins, thereby perpetuating inflammatory signaling and vascular injury even after viral clearance, may activate the lectin complement pathway. These observations support the concept that cytokine signaling and complement activation act synergistically to sustain chronic inflammation and multisystem dysfunction [10, 11].

Persistent viral reservoirs may provide continuous immune stimulation that maintains both cytokine production and complement activation. SARS‐CoV‐2 proteins have been detected in the gastrointestinal tract, lymphoid tissues, and other organs months after acute infection, where they may stimulate macrophages, dendritic cells, and complement pathways through pattern‐recognition receptors and lectin pathway activation. Viral spike and nucleocapsid proteins have also been shown to directly activate complement, providing a mechanistic link between antigen persistence, endothelial injury, and chronic thromboinflammation. These findings suggest that viral persistence, complement activation, and cytokine dysregulation are interconnected rather than independent processes [12]. Endothelial dysfunction has emerged as another hallmark of Long COVID. Persistent endothelial activation, characterized by increased expression of adhesion molecules, von Willebrand factor, angiopoietin‐2, and procoagulant mediators, contributes to microvascular dysfunction, impaired tissue perfusion, and ongoing organ injury. Complement‐mediated endothelial damage further amplifies cytokine production, platelet activation, and coagulation abnormalities, establishing a self‐perpetuating cycle of inflammation and vascular dysfunction. This integrated model helps explain the diverse clinical manifestations observed in Long COVID, including neurological, cardiovascular, pulmonary, and systemic symptoms [13].

Advances in systems immunology, single‐cell transcriptomics, proteomics, metabolomics, spatial biology, and artificial intelligence (AI)‐assisted multi‐omics analyses have substantially improved understanding of the molecular heterogeneity of Long COVID. These technologies have facilitated the identification of distinct inflammatory endotypes and biomarker signatures that may enable diagnosis that is more accurate, prognostic stratification, and individualized therapeutic selection. At the same time, several targeted immunomodulatory approaches—including cytokine inhibitors, complement inhibitors, Janus kinase (JAK) inhibitors, mesenchymal stem cell therapy, microbiome‐directed interventions, and antifibrotic therapies—are being actively investigated as potential treatments for selected Long COVID phenotypes [12, 13]. The aim of this narrative review is to comprehensively examine the current understanding of cytokine‐ and complement‐driven hyperinflammation in Long COVID, with particular emphasis on the molecular mechanisms underlying persistent immune dysregulation, emerging inflammatory and vascular biomarkers, and evolving immunotherapeutic strategies. In addition, the review discusses recent advances in precision medicine, multi‐omics technologies, and AI‐assisted biomarker discovery that may facilitate personalized management and improve long‐term outcomes for patients with Long COVID.

## 2 Methods

This manuscript was prepared as a narrative review aimed at synthesizing current evidence on cytokine‐driven hyperinflammation in chronic COVID, with a focus on mechanistic pathways, biomarkers, and emerging immunotherapies. The literature search was conducted across PubMed, Scopus, and Web of Science databases, covering articles published from January 2020 to December 2025. Search terms included combinations of “chronic COVID,” “long COVID,” “post‐acute sequelae of SARS‐CoV‐2,” “cytokines,” “hyperinflammation,” “immune dysregulation,” “biomarkers,” and “immunotherapy.” Inclusion criteria were: (1) original research studies, reviews, meta‐analyses, or clinical trials addressing immunological mechanisms in chronic COVID; (2) studies reporting cytokine profiles, immune‐cell alterations, or multi‐omics findings; (3) reports on immunomodulatory therapies in the context of chronic COVID or prolonged post‐infectious inflammation. Exclusion criteria included non‐English publications, case reports with fewer than five participants, and studies focused solely on acute COVID without relevant chronic‐phase data.

The selection process involved initial screening of titles and abstracts, followed by full‐text review to ensure relevance and methodological quality. Key studies were evaluated for study design, cohort characteristics, sample size, immunological assays, and outcome measures. Special attention was given to studies that distinguished acute‐phase from chronic‐phase immune responses. Data synthesis emphasized thematic analysis, comparison of mechanistic pathways, identification of knowledge gaps, and translation of biomarkers into potential patient‐stratification frameworks. Where available, findings from multicenter cohorts and randomized trials were prioritized to strengthen the validity and generalizability of the conclusions. The narrative approach allowed integration of heterogeneous data, highlighting both consistent trends and areas of controversy in the current literature. Figures and tables were generated to summarize cytokine patterns, immune‐cell alterations, and potential therapeutic targets across acute and chronic COVID, facilitating clear comparisons and translational relevance.

### 2.1 Gaps, Inconsistencies, and Limitations in Current Immunological Data

Despite substantial progress in understanding the immunopathogenesis of Long COVID, significant gaps remain regarding the mechanisms responsible for persistent symptoms and the identification of reliable therapeutic targets. Long COVID is increasingly recognized as a heterogeneous syndrome comprising multiple biological endotypes rather than a single disease entity. Consequently, considerable variability exists in reported immunological findings across studies, limiting the translation of research discoveries into routine clinical practice. One of the major challenges is the inconsistency in cytokine profiling. While persistent elevations of interleukin (IL)‐6, IL‐1β, tumor necrosis factor‐alpha (TNF‐α), interferon‐gamma (IFN‐γ), IL‐17, and several chemokines have been reported, other investigations have observed only modest changes or no significant differences compared with recovered individuals. These discrepancies likely reflect differences in patient populations, disease severity during acute infection, vaccination status, circulating viral variants, and timing of sample collection, laboratory methodologies, and definitions of Long COVID. Moreover, circulating cytokine concentrations may not accurately represent localized tissue inflammation occurring within the lungs, central nervous system, gastrointestinal tract, or cardiovascular system.

Similarly, although persistent activation of the alternative and lectin complement pathways has emerged as an important feature of Long COVID, the prevalence, duration, and clinical significance of complement dysregulation remain incompletely defined. Current evidence suggests that complement activation contributes to endothelial injury, thromboinflammation, and microvascular dysfunction; however, it is uncertain whether complement activation represents a primary pathogenic mechanism or a downstream consequence of persistent viral antigen exposure and chronic immune activation. Standardized assays for measuring complement activation products are also lacking, limiting comparisons between studies. Another important limitation is the absence of universally accepted diagnostic biomarkers. Most currently proposed biomarkers—including cytokines, complement proteins, endothelial activation markers, coagulation parameters, and autoantibodies—lack sufficient sensitivity and specificity for routine clinical diagnosis or disease monitoring. Furthermore, many studies are cross‐sectional, involve relatively small cohorts, or lack appropriate control groups, thereby limiting causal inference and generalizability. Longitudinal investigations that characterize immune trajectories from acute infection through recovery and Long COVID are still relatively scarce.

The contribution of persistent viral reservoirs remains controversial. Although SARS‐CoV‐2 RNA and viral proteins have been detected in gastrointestinal tissue, lymphoid organs, and other anatomical sites months after infection, the frequency, biological relevance, and relationship between viral persistence, complement activation, and sustained cytokine production require further clarification. Likewise, the interactions among viral persistence, latent virus reactivation, gut microbiome dysbiosis, mitochondrial dysfunction, and autoimmunity are incompletely understood and likely differ among clinical phenotypes. Therapeutically, robust evidence supporting immunomodulatory interventions remains limited. Most cytokine‐targeted therapies, complement inhibitors, primarily early‐phase studies, observational cohorts, or extrapolation from acute COVID‐19 and other inflammatory diseases support Janus kinase inhibitors, mesenchymal stem cell therapies, microbiome‐directed interventions, and antifibrotic agents. Large, multicenter randomized controlled trials with biomarker‐guided patient selection are needed to determine which patients are most likely to benefit from specific immunotherapeutic approaches while minimizing unnecessary immunosuppression. Finally, advances in multi‐omics technologies, single‐cell sequencing, spatial transcriptomics, and AI offer unprecedented opportunities to characterize the biological heterogeneity of Long COVID. However, these approaches are challenged by limited external validation, heterogeneous datasets, and lack of standardized analytical pipelines, algorithmic bias, and restricted access to high‐quality multicenter data. Future research should prioritize harmonized definitions of Long COVID, standardized immune phenotyping protocols, validated biomarker panels, and integrative multi‐omics studies linked to prospective clinical outcomes. Such efforts will be essential for developing precision medicine strategies that accurately identify disease endotypes, predict therapeutic responses, and improve long‐term management of individuals living with Long COVID.

### 2.2 Pathophysiology of Cytokine‐ and Complement‐Mediated Hyperinflammation in Long COVID

Long COVID is increasingly recognized as a complex immunopathological syndrome resulting from persistent interactions among viral antigen persistence, dysregulated innate and adaptive immunity, complement activation, endothelial dysfunction, thromboinflammation, and impaired tissue repair. Rather than reflecting a single inflammatory pathway, persistent symptoms appear to arise from interconnected biological processes that vary across clinical phenotypes. Current evidence suggests that cytokine‐mediated inflammation and complement dysregulation act synergistically to sustain chronic immune activation, microvascular injury, and multisystem dysfunction long after the resolution of acute SARS‐CoV‐2 infection [14]. One of the principal mechanisms proposed for Long COVID is the persistence of viral reservoirs or viral antigens within tissues such as the gastrointestinal tract, lymphoid organs, central nervous system, and cardiovascular tissues. Although replication‐competent virus is rarely detected months after infection, persistent spike and nucleocapsid proteins may continue to stimulate innate immune receptors, including Toll‐like receptors and nucleotide‐binding oligomerization domain‐like receptors, thereby maintaining activation of macrophages, dendritic cells, and monocytes. Persistent viral proteins have also been shown to activate the lectin complement pathway, providing an additional mechanism for sustained inflammation and vascular injury [15].

Chronic activation of innate immune cells leads to prolonged production of pro‐inflammatory cytokines, including interleukin (IL)‐6, IL‐1β, tumor necrosis factor‐alpha (TNF‐α), interferon‐gamma (IFN‐γ), IL‐17, and chemokines such as CXCL9, CXCL10, CCL2, and CXCL8. IL‐6 promotes hepatic acute‐phase responses, endothelial activation, B‐cell maturation, and neuroinflammation, whereas IL‐1β amplifies inflammasome signaling and contributes to persistent systemic inflammation and tissue injury. TNF‐α further enhances endothelial dysfunction, oxidative stress, insulin resistance, and skeletal muscle catabolism, while IFN‐γ sustains macrophage activation and persistent immune stimulation. Increased IL‐17 production by T helper 17 cells has also been associated with chronic inflammation, pulmonary fibrosis, and autoimmune phenomena observed in subsets of patients with Long COVID [16, 17]. Complement activation has emerged as another major contributor to disease pathogenesis. Recent proteomic and immunological studies have demonstrated persistent activation of the alternative complement pathway together with evidence of ongoing thromboinflammation months after acute infection. Elevated levels of complement activation products, including C3a, C5a, soluble C5b‐9, and factor Bb, have been associated with endothelial injury, platelet activation, leukocyte recruitment, and impaired fibrinolysis. Activation of the lectin pathway by SARS‐CoV‐2 structural proteins further amplifies inflammatory cascades through generation of potent anaphylatoxins and membrane attack complexes, creating a positive feedback loop between complement activation and cytokine production [18].

The interaction between cytokines and complement contributes directly to endothelial dysfunction, which is now recognized as a hallmark of Long COVID. Activated endothelial cells exhibit increased expression of vascular cell adhesion molecule‐1 (VCAM‐1), intercellular adhesion molecule‐1 (ICAM‐1), von Willebrand factor, angiopoietin‐2, and tissue factor, promoting leukocyte adhesion, platelet aggregation, coagulation activation, and microvascular thrombosis. Persistent endothelial injury impairs tissue perfusion and oxygen delivery, potentially contributing to fatigue, exercise intolerance, cognitive dysfunction, and cardiovascular complications frequently reported in Long COVID [19]. Adaptive immune dysregulation further perpetuates chronic inflammation. Several studies have reported persistent activation and exhaustion of CD4^+^ and CD8^+^ T lymphocytes, reduced naïve T‐cell populations, increased expression of inhibitory receptors such as programmed cell death protein 1 (PD‐1) and T‐cell immunoglobulin and mucin‐domain containing‐3 (TIM‐3), and expansion of activated memory T cells. B‐cell abnormalities, including persistent plasmablast activation and autoantibody production, have also been described, supporting the hypothesis that autoimmune mechanisms contribute to persistent symptoms in a subset of patients. These alterations may be driven by continued antigen exposure, molecular mimicry, or dysregulated immune tolerance [20].

**Figure 1:**
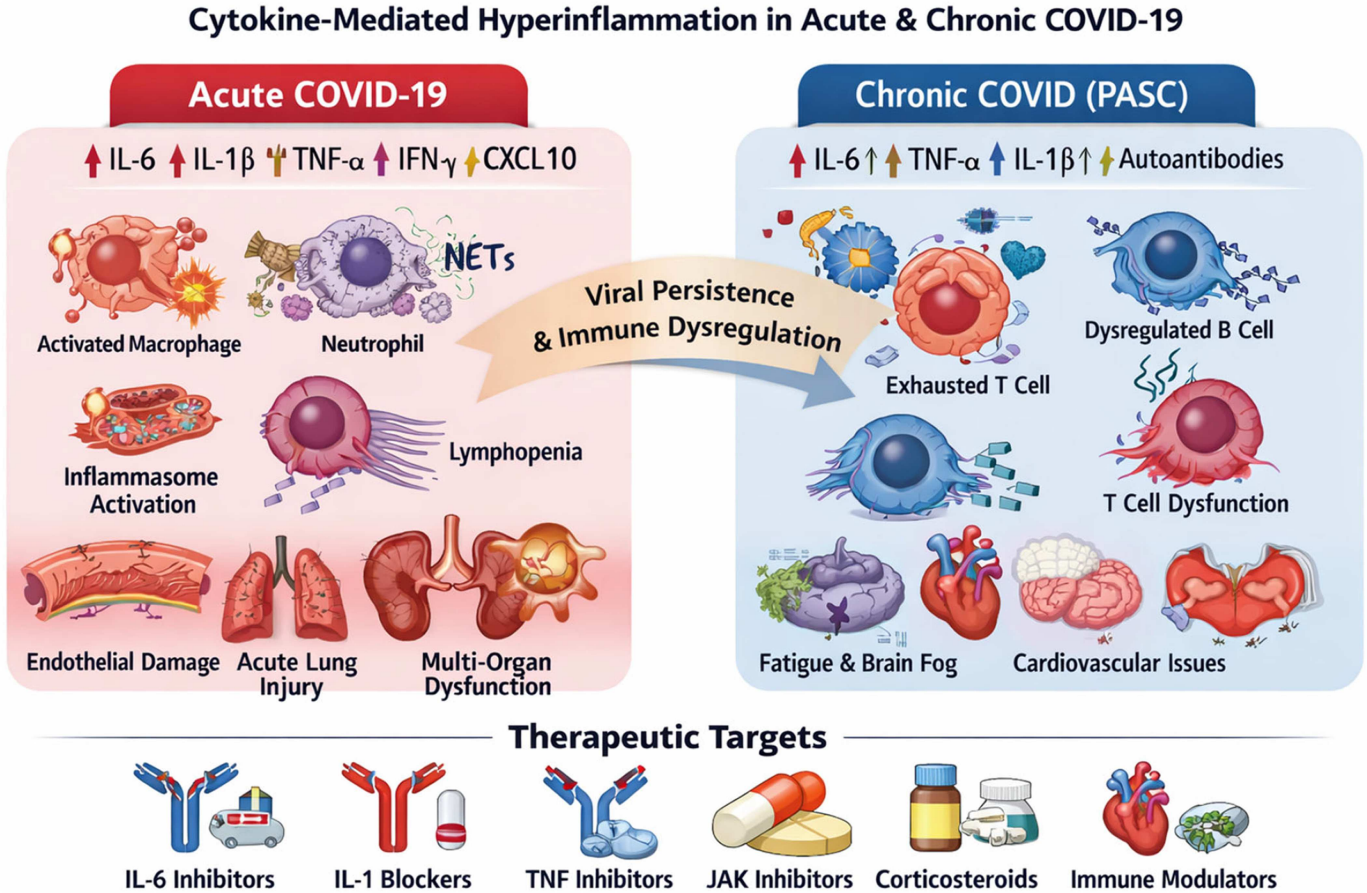
Cytokine‐mediated hyperinflammation in acute and chronic COVID‐19.

Neuroinflammation represents another important component of Long COVID pathophysiology. Pro‐inflammatory cytokines, complement proteins, activated monocytes, and circulating immune complexes may disrupt blood–brain barrier integrity and activate microglia and astrocytes, resulting in persistent neuroinflammation. These mechanisms have been implicated in cognitive impairment, memory dysfunction, headache, sleep disturbances, anxiety, depression, and autonomic dysfunction. In parallel, chronic inflammation contributes to mitochondrial dysfunction and oxidative stress, reducing cellular energy production and potentially explaining the profound fatigue and post‐exertional symptom exacerbation experienced by many patients [19]. Additional mechanisms likely interact with cytokine and complement dysregulation to sustain chronic disease. These include reactivation of latent viruses such as Epstein–Barr virus, alterations of the gut microbiome with increased intestinal permeability, metabolic reprogramming of immune cells, persistent platelet activation, and impaired fibrinolysis leading to fibrin‐rich microclots. These interconnected pathways establish a self‐perpetuating cycle of inflammation, endothelial dysfunction, immune activation, and tissue remodeling that may continue for months or years after acute infection (Figure 1) [20].

### 2.3 Mechanistic Pathways of Cytokine‐ and Complement‐Driven Hyperinflammation in Long COVID

The pathogenesis of Long COVID is increasingly understood as a dynamic network of interconnected inflammatory pathways rather than the consequence of a single immune abnormality. Persistent viral antigen stimulation, dysregulated innate and adaptive immune responses, complement activation, endothelial dysfunction, thromboinflammation, and impaired tissue repair collectively establish a chronic inflammatory state that underlies the diverse clinical manifestations of the syndrome. These mechanisms interact through multiple positive feedback loops, sustaining immune activation long after the resolution of acute SARS‐CoV‐2 infection [21].

## 3 Persistent Viral Antigen Stimulation

One of the leading hypotheses for Long COVID is the persistence of viral antigens or viral reservoirs within tissues, including the gastrointestinal tract, lymphoid organs, central nervous system, and cardiovascular tissues. Although infectious virus is rarely detected during the post‐acute phase, persistent spike and nucleocapsid proteins may continuously stimulate pattern‐recognition receptors such as toll‐like receptors (TLRs), retinoic acid‐inducible gene‐I (RIG‐I)‐like receptors, and nucleotide‐binding oligomerization domain (NOD)‐like receptors. This sustained innate immune activation promotes prolonged secretion of inflammatory cytokines while also activating the lectin complement pathway. Persistent antigen exposure may therefore represent the initiating event that links viral persistence with chronic inflammation, endothelial injury, and immune dysregulation [22].

## 4 Innate Immune Activation and Cytokine Amplification

Persistent activation of monocytes, macrophages, dendritic cells, and neutrophils contributes substantially to the maintenance of chronic inflammation. Activated innate immune cells continuously produce pro‐inflammatory mediators, including interleukin (IL)‐6, IL‐1β, tumor necrosis factor‐alpha (TNF‐α), interferon‐gamma (IFN‐γ), IL‐17, IL‐8, CXCL9, CXCL10, and CCL2. IL‐6 promotes hepatic acute‐phase protein synthesis, endothelial activation, B‐cell differentiation, and neuroinflammation, whereas IL‐1β, generated through activation of the NLRP3 inflammasome, amplifies inflammatory signaling and tissue injury. TNF‐α enhances oxidative stress, vascular dysfunction, and skeletal muscle catabolism, while IFN‐γ sustains macrophage activation and adaptive immune responses. Collectively, these cytokines establish a persistent inflammatory milieu that contributes to fatigue, cognitive impairment, pulmonary dysfunction, and systemic symptoms [23].

## 5 Complement Activation and Thromboinflammation

Complement dysregulation has emerged as a central mechanism linking immune activation with vascular pathology in Long COVID. Recent proteomic studies have demonstrated persistent activation of the alternative complement pathway, accompanied by elevated levels of complement activation products such as C3a, C5a, soluble C5b‐9, and factor Bb. SARS‐CoV‐2 spike and nucleocapsid proteins can also activate the lectin pathway through mannose‐binding lectin‐associated serine proteases, resulting in amplification of complement‐mediated inflammation. Complement activation generates potent anaphylatoxins that recruit leukocytes, activate platelets, increase endothelial permeability, and stimulate additional cytokine release, thereby establishing reciprocal amplification between complement and cytokine signaling pathways [24].

## 6 Endothelial Dysfunction and Microvascular Injury

Persistent inflammation and complement activation contribute directly to endothelial injury, a defining feature of Long COVID. Activated endothelial cells exhibit increased expression of vascular cell adhesion molecule‐1 (VCAM‐1), intercellular adhesion molecule‐1 (ICAM‐1), E‐selectin, von Willebrand factor, angiopoietin‐2, and tissue factor. These changes promote leukocyte adhesion, platelet aggregation, activation of coagulation pathways, and impaired fibrinolysis, leading to microvascular thrombosis and reduced tissue perfusion. Chronic endothelial dysfunction has been implicated in exercise intolerance, dysautonomia, cognitive dysfunction, cardiopulmonary complications, and persistent fatigue [25].

## 7 Adaptive Immune Dysregulation

Long COVID is associated with sustained abnormalities of adaptive immunity. Persistent activation and exhaustion of CD4^+^ and CD8^+^ T lymphocytes, reduced naïve T‐cell populations, increased expression of inhibitory receptors such as programmed cell death protein‐1 (PD‐1) and T‐cell immunoglobulin and mucin‐domain containing‐3 (TIM‐3), and expansion of activated memory T cells have been consistently reported. B‐cell dysregulation, including persistent plasmablast activation and autoantibody production, further contributes to chronic immune activation and may explain autoimmune manifestations observed in some patients. These adaptive immune abnormalities perpetuate cytokine production and prolong inflammatory responses even after viral clearance [26].

## 8 Neuroimmune and Mitochondrial Dysfunction

Persistent systemic inflammation affects the central nervous system through disruption of the blood–brain barrier, activation of microglia and astrocytes, and infiltration of circulating inflammatory mediators. Cytokines such as IL‐6, TNF‐α, IFN‐γ, and complement‐derived anaphylatoxins contribute to neuroinflammation, which has been associated with cognitive dysfunction, memory impairment, headache, sleep disturbances, depression, anxiety, and autonomic dysfunction. Simultaneously, chronic inflammation induces mitochondrial dysfunction, oxidative stress, and altered cellular metabolism, reducing adenosine triphosphate production and contributing to fatigue and post‐exertional symptom exacerbation [27].

## 9 Fibrosis, Tissue Remodeling, and Organ Dysfunction

Persistent inflammatory signaling also promotes fibroblast activation and extracellular matrix remodeling. Transforming growth factor‐beta (TGF‐β), IL‐17, TNF‐α, and complement‐mediated inflammatory pathways stimulate collagen deposition and tissue fibrosis within the lungs, myocardium, kidneys, and other organs. Progressive tissue remodeling may contribute to persistent respiratory impairment, reduced exercise capacity, cardiovascular dysfunction, and chronic organ‐specific sequelae in susceptible individuals [28].

## 10 Integrated Immunopathological Model

Current evidence supports an integrated model in which persistent viral antigens initiate chronic innate immune activation, sustained cytokine production amplifies inflammatory signaling, complement activation promotes thromboinflammation and endothelial injury, adaptive immune dysregulation perpetuates immune activation, and mitochondrial dysfunction and fibrosis contribute to long‐term organ dysfunction. Rather than acting independently, these pathways reinforce one another through multiple positive feedback mechanisms, generating distinct biological endotypes that likely explain the clinical heterogeneity of Long COVID. Recognizing these interconnected mechanistic pathways provides a strong biological foundation for precision medicine approaches based on immune phenotyping, biomarker‐guided risk stratification, and targeted therapeutic interventions. Future studies integrating longitudinal immune profiling, multi‐omics technologies, and AI‐driven analyses will be essential for identifying clinically actionable inflammatory signatures and optimizing personalized treatment strategies for individuals with Long COVID [28].

### 10.1 Comparative Immunological Evidence from Acute COVID‐19 and Long COVID Cohorts

Comparative analyses of immune responses during acute COVID‐19 and Long COVID have substantially improved understanding of the transition from acute viral infection to persistent multisystem disease. Although both conditions involve immune activation, they differ markedly in the magnitude, duration, and biological characteristics of inflammatory responses. Acute COVID‐19 is characterized by robust antiviral immunity that, in severe cases, may progress to excessive systemic inflammation, whereas Long COVID is associated with sustained, low‐grade immune activation, persistent complement dysregulation, endothelial dysfunction, and altered immune homeostasis that may persist for months after the initial infection [29]. During acute COVID‐19, rapid activation of the innate immune system leads to increased production of pro‐inflammatory cytokines, including interleukin (IL)‐6, IL‐1β, tumor necrosis factor‐alpha (TNF‐α), interferon‐gamma (IFN‐γ), IL‐8, and granulocyte‐macrophage colony‐stimulating factor (GM‐CSF). In severe disease, these mediators contribute to diffuse endothelial injury, vascular permeability, coagulation abnormalities, acute respiratory distress syndrome, and multiorgan dysfunction. However, subsequent investigations have demonstrated that severe COVID‐19 is not solely a consequence of a “cytokine storm.” Complement activation, particularly through the alternative and lectin pathways, plays a pivotal role in endothelial injury, platelet activation, thromboinflammation, and microvascular thrombosis, working in concert with cytokine‐mediated inflammatory pathways rather than independently [30].

In contrast, Long COVID is characterized by persistent immune activation despite clinical recovery from acute infection. Longitudinal cohort studies have demonstrated sustained elevations of IL‐6, IL‐1β, TNF‐α, IFN‐γ, IL‐17, CXCL9, CXCL10, CCL2, and other inflammatory mediators in subsets of patients months after infection. Although cytokine concentrations are generally lower than those observed during severe acute disease, their prolonged elevation appears sufficient to maintain chronic inflammation, neuroimmune dysfunction, endothelial activation, and impaired tissue repair. Importantly, not all individuals with Long COVID exhibit identical cytokine profiles, reflecting substantial biological heterogeneity and the likelihood of multiple immunological endotypes [31]. Recent proteomic and immunological investigations have identified persistent complement dysregulation as one of the distinguishing immunological features of Long COVID. Longitudinal studies have demonstrated sustained activation of the alternative complement pathway, persistent elevation of complement activation products such as C3a, C5a, soluble C5b‐9, and factor Bb, together with evidence of ongoing thromboinflammation, platelet activation, and impaired fibrinolysis. Persistent activation of the lectin pathway has also been implicated through interactions between residual SARS‐CoV‐2 proteins and mannose‐binding lectin‐associated serine proteases. These findings suggest that complement‐mediated vascular injury continues beyond the acute phase and may contribute directly to persistent fatigue, exercise intolerance, cognitive dysfunction, and cardiopulmonary symptoms [32].

Differences in adaptive immune responses further distinguish acute COVID‐19 from Long COVID. Acute infection is typically associated with vigorous activation of virus‐specific T and B‐lymphocytes followed by gradual immune resolution in most individuals. Conversely, Long COVID cohorts frequently demonstrate persistent activation and exhaustion of CD4^+^ and CD8^+^ T cells, reduced naïve T‐cell populations, increased expression of inhibitory receptors such as programmed cell death protein‐1 (PD‐1), T‐cell immunoglobulin, and mucin‐domain containing‐3 (TIM‐3), expansion of activated memory T cells, and sustained plasmablast responses. Autoantibody production has also been reported in subsets of patients, suggesting that dysregulated adaptive immunity and loss of immune tolerance may contribute to prolonged symptomatology [33]. Endothelial dysfunction represents another important distinction between acute and persistent disease. During acute COVID‐19, endothelial injury results primarily from direct viral effects, inflammatory cytokines, complement activation, and coagulation abnormalities. In Long COVID, persistent endothelial activation is characterized by elevated concentrations of vascular cell adhesion molecule‐1 (VCAM‐1), intercellular adhesion molecule‐1 (ICAM‐1), von Willebrand factor, angiopoietin‐2, and tissue factor, reflecting ongoing vascular inflammation and impaired microcirculatory function despite apparent clinical recovery. Persistent endothelial dysfunction may explain the high prevalence of cardiovascular symptoms, dysautonomia, and exercise intolerance observed in Long COVID [31]. Comparative multi‐omics studies have further demonstrated that Long COVID exhibits distinctive transcriptomic, proteomic, metabolomic, and immunophenotypic signatures that differ substantially from those observed during acute infection. Persistent activation of inflammatory, coagulation, complement, and fibrosis‐related pathways has been accompanied by evidence of mitochondrial dysfunction, altered lipid metabolism, gut microbiome dysbiosis, and impaired cellular bioenergetics. These molecular alterations support the concept that Long COVID represents a state of chronic immune dysregulation rather than incomplete recovery from acute disease [32, 33] (Table 1).

**Table 1:** Comparative immunological features of acute COVID‐19 and long COVID (post‐acute sequelae of SARS‐CoV‐2 infection).

Feature	Acute COVID‐19	Long COVID (PASC)
Disease phase	Active SARS‐CoV‐2 infection with acute viral replication	Persistent or newly emerging symptoms ≥ 3 months after acute infection
Dominant immunopathology	Acute antiviral immune response with hyperinflammation in severe disease	Persistent low‐grade immune dysregulation with chronic inflammation and immune imbalance
Viral status	Active viral replication	Persistent viral antigens and/or tissue reservoirs proposed in a subset of patients; replication‐competent virus is rarely detected
Cytokine profile	Marked elevation of IL‐6, IL‐1β, TNF‐α, IFN‐γ, IL‐8, GM‐CSF, and other inflammatory mediators, particularly in severe disease	Persistent but generally lower elevations of IL‐6, IL‐1β, TNF‐α, IFN‐γ, IL‐17, CXCL9, CXCL10, CCL2, and related cytokines/chemokines
Complement activation	Robust activation of the classical, lectin, and alternative pathways contributes to endothelial injury, thromboinflammation, and multiorgan damage	Sustained activation of the alternative and lectin pathways with persistent elevations of C3a, C5a, soluble C5b‐9, and factor Bb, contributing to chronic vascular inflammation
Innate immune response	Rapid activation of monocytes, macrophages, neutrophils, dendritic cells, and natural killer cells	Persistent monocyte/macrophage activation, altered dendritic cell function, reduced natural killer cell cytotoxicity, and chronic innate immune activation
Adaptive immune response	Robust virus‐specific T‐ and B‐cell activation followed by immune contraction in most patients	Persistent T‐cell activation and exhaustion, reduced naïve T‐cell populations, activated memory T cells, plasmablast expansion, and autoantibody production in selected patients
Inflammasome activity	Marked NLRP3 inflammasome activation during severe disease	Persistent low‐grade inflammasome activation contributing to chronic IL‐1β production and tissue injury
Endothelial dysfunction	Acute endothelial injury, increased vascular permeability, platelet activation, and coagulation abnormalities	Persistent endothelial activation with elevated VCAM‐1, ICAM‐1, von Willebrand factor, angiopoietin‐2, and ongoing microvascular dysfunction
Coagulation abnormalities	Hypercoagulability, disseminated intravascular coagulation in severe cases, venous and arterial thrombosis	Persistent thromboinflammation, elevated d‐dimer, impaired fibrinolysis, platelet activation, and possible fibrin‐rich microclots in selected patients
Neuroimmune involvement	Acute encephalopathy and systemic neuroinflammation in severe disease	Chronic neuroinflammation associated with cognitive dysfunction, fatigue, headache, dysautonomia, sleep disturbances, and mood disorders
Fibrosis and tissue remodeling	Early tissue injury with potential progression to fibrosis in severe disease	Persistent activation of TGF‐β‐mediated fibrotic pathways contributing to pulmonary and extrapulmonary tissue remodeling
Autoimmunity	Autoantibodies reported in severe acute infection	Persistent autoantibody production and immune dysregulation observed in subsets of patients
Major biomarkers	CRP, ferritin, IL‐6, IL‐1β, TNF‐α, d‐dimer, lactate dehydrogenase, ferritin, complement activation products	IL‐6, IL‐1β, TNF‐α, IL‐17, CXCL10, C3a, C5a, soluble C5b‐9, factor Bb, VCAM‐1, ICAM‐1, von Willebrand factor, d‐dimer, multi‐omics signatures
Dominant pathological mechanisms	Viral cytopathic effects, cytokine‐mediated inflammation, complement activation, endothelial injury, and thromboinflammation	Persistent cytokine signaling, complement dysregulation, endothelial dysfunction, viral antigen persistence, immune exhaustion, autoimmunity, mitochondrial dysfunction, and chronic thromboinflammation
Principal therapeutic approaches	Antivirals, corticosteroids, IL‐6 inhibitors, JAK inhibitors, anticoagulation, supportive care, selected complement inhibitors under investigation	Symptom‐directed care, rehabilitation, cytokine‐targeted biologics, complement inhibitors, JAK inhibitors, microbiome‐directed therapies, mesenchymal stem cell therapy, antifibrotic agents, and biomarker‐guided precision immunotherapy (largely investigational)

Abbreviations: C3a, complement component 3a; C5a, complement component 5a; C5b‐9, terminal complement complex; CCL2, C‐C motif chemokine ligand 2; CRP, C‐reactive protein; CXCL9/CXCL10, C‐X‐C motif chemokine ligands 9 and 10; GM‐CSF, granulocyte‐macrophage colony‐stimulating factor; ICAM‐1, intercellular adhesion molecule‐1; IFN‐γ, interferon‐gamma; IL, interleukin; JAK, Janus kinase; PASC, post‐acute sequelae of SARS‐CoV‐2 infection; TGF‐β, transforming growth factor‐beta; TNF‐α, tumor necrosis factor‐alpha; VCAM‐1, vascular cell adhesion molecule‐1.

#### 10.1.1 Biomarkers and Multi‐Omics Insights in Long Covid

The identification of reliable biomarkers remains a major priority in Long COVID research because of the heterogeneous clinical presentation and the absence of universally accepted diagnostic tests. Current evidence indicates that no single biomarker adequately captures the complexity of Long COVID. Instead, combinations of inflammatory, immunological, endothelial, coagulation, metabolic, and tissue injury biomarkers, integrated with multi‐omics technologies, offer greater potential for disease characterization, patient stratification, and therapeutic monitoring. These approaches are increasingly revealing distinct biological endotypes that may support precision medicine in Long COVID.

## 11 Inflammatory and Cytokine Biomarkers

Persistent immune activation is reflected by sustained elevations of several circulating cytokines and chemokines. Among the most consistently reported biomarkers are interleukin (IL)‐6, IL‐1β, tumor necrosis factor‐alpha (TNF‐α), interferon‐gamma (IFN‐γ), IL‐17, IL‐8, CXCL9, CXCL10, and CCL2. Elevated IL‐6 has been associated with fatigue, impaired exercise tolerance, and cognitive dysfunction, while IL‐1β reflects ongoing inflammasome activation and chronic innate immune stimulation. TNF‐α contributes to endothelial dysfunction, muscle catabolism, and persistent systemic inflammation, whereas IL‐17 has been linked to pulmonary fibrosis, chronic respiratory symptoms, and autoimmune manifestations. Although these cytokines provide important mechanistic insights, considerable variability between studies highlights the need for standardized sampling protocols and longitudinal validation before routine clinical implementation [34].

## 12 Complement Biomarkers

Persistent complement activation has emerged as one of the most promising biological signatures of Long COVID. Elevated circulating concentrations of C3a, C5a, soluble C5b‐9, factor Bb, and other complement activation products have been associated with endothelial injury, platelet activation, thromboinflammation, and impaired fibrinolysis. Proteomic studies have demonstrated sustained activation of the alternative complement pathway months after acute infection, while increasing evidence also implicates activation of the lectin pathway by persistent SARS‐CoV‐2 structural proteins. These findings suggest that complement biomarkers may improve identification of patients with ongoing vascular inflammation and could facilitate selection of individuals most likely to benefit from complement‐targeted therapies [35].

## 13 Endothelial and Vascular Biomarkers

Endothelial dysfunction represents a central feature of Long COVID and is reflected by persistent elevations of vascular cell adhesion molecule‐1 (VCAM‐1), intercellular adhesion molecule‐1 (ICAM‐1), E‐selectin, von Willebrand factor (vWF), angiopoietin‐2, soluble thrombomodulin, and endothelin‐1. These biomarkers indicate sustained endothelial activation, increased vascular permeability, platelet adhesion, and impaired microvascular integrity. Persistent endothelial injury has been correlated with exercise intolerance, dysautonomia, cardiovascular complications, and neurovascular dysfunction, emphasizing the importance of vascular biomarkers in disease monitoring [36].

## 14 Coagulation and Thromboinflammatory Biomarkers

Chronic activation of coagulation pathways remains evident in many patients with Long COVID. Elevated d‐dimer, fibrinogen, thrombin–antithrombin complexes, plasminogen activator inhibitor‐1 (PAI‐1), fibrin degradation products, and platelet activation markers support the presence of persistent thromboinflammation and impaired fibrinolysis. The detection of fibrin amyloid microclots in selected patient cohorts has further strengthened the hypothesis that persistent microvascular obstruction contributes to ongoing tissue hypoxia and multisystem symptoms, although additional validation is required before incorporation into routine clinical practice [37].

## 15 Immune Cell and Autoimmune Biomarkers

Advanced immunophenotyping has identified persistent alterations in both innate and adaptive immune cell populations. Long COVID has been associated with activated monocytes, reduced natural killer cell cytotoxicity, persistent plasmablast expansion, exhausted CD4^+^ and CD8^+^ T cells expressing programmed cell death protein‐1 (PD‐1) and T‐cell immunoglobulin and mucin‐domain containing‐3 (TIM‐3), and expansion of activated memory T‐cell subsets. Autoantibodies directed against nuclear, endothelial, adrenergic, muscarinic, and phospholipid antigens have also been reported in subsets of patients, suggesting that autoimmune mechanisms may contribute to disease persistence in specific immunological endotypes [38].

## 16 Multi‐Omics Technologies

Recent advances in multi‐omics have transformed understanding of the molecular complexity of Long COVID. Transcriptomic studies have demonstrated persistent activation of inflammatory, interferon, complement, and coagulation‐related gene networks. Single‐cell RNA sequencing has revealed sustained activation of monocytes, macrophages, endothelial cells, and exhausted lymphocyte populations while identifying cell‐type‐specific inflammatory programs that are not detectable using conventional bulk analyses. Proteomic investigations have consistently demonstrated persistent abnormalities involving complement proteins, coagulation factors, inflammatory mediators, extracellular matrix remodeling proteins, and markers of endothelial dysfunction. These studies have identified proteomic signatures that distinguish individuals with Long COVID from fully recovered controls and suggest potential therapeutic targets. Metabolomic analyses have revealed alterations in amino acid metabolism, lipid metabolism, mitochondrial energy production, oxidative stress pathways, and tricarboxylic acid cycle intermediates, supporting the hypothesis that impaired cellular bioenergetics contributes to fatigue and exercise intolerance. Lipidomic studies have further demonstrated persistent disturbances in phospholipid metabolism, specialized pro‐resolving mediators, and inflammatory lipid signaling pathways. Emerging microbiome studies have identified reduced intestinal microbial diversity, depletion of beneficial commensal bacteria, and enrichment of proinflammatory microbial taxa. These alterations may contribute to systemic immune activation through increased intestinal permeability, microbial translocation, and disruption of the gut–lung–brain axis. Integration of microbiome profiles with inflammatory biomarkers may improve understanding of disease heterogeneity and identify novel therapeutic opportunities [37, 38].

## 17 AI and Integrated Multi‐Omics

AI and machine learning have become increasingly valuable for integrating high‐dimensional clinical, laboratory, imaging, and multi‐omics datasets. These computational approaches facilitate identification of complex biomarker signatures that outperform individual biomarkers for diagnosing Long COVID, predicting disease severity, and identifying biologically distinct inflammatory endotypes. Explainable AI models may also assist in selecting patients for targeted immunotherapies based on individualized molecular profiles. However, broader clinical implementation requires standardized data collection, external validation across diverse populations, transparent algorithms, and harmonized analytical frameworks to minimize bias and improve reproducibility [38].

### 17.1 Immunomodulatory Therapeutic Strategies

The heterogeneous immunopathology of Long COVID suggests that no single therapeutic approach is likely to be universally effective. Instead, treatment strategies should ideally target the dominant pathogenic mechanisms present within individual patients, including persistent cytokine‐mediated inflammation, complement dysregulation, endothelial dysfunction, thromboinflammation, viral antigen persistence, autoimmunity, and tissue remodeling. Although no immunomodulatory therapy has yet been approved specifically for Long COVID, several targeted interventions are being investigated based on mechanistic insights gained from acute COVID‐19, autoimmune diseases, and chronic inflammatory disorders. Current evidence remains preliminary, and most therapeutic approaches require confirmation in adequately powered randomized controlled trials [12]. Persistent elevation of proinflammatory cytokines has prompted investigation of biologic agents that selectively inhibit inflammatory signaling pathways. Interleukin‐6 (IL‐6) inhibitors, including tocilizumab and sarilumab, have demonstrated clinical benefit in selected patients with severe acute COVID‐19 and are being explored for individuals with persistent inflammatory phenotypes of Long COVID. By inhibiting IL‐6 receptor signaling, these agents may reduce systemic inflammation, endothelial activation, neuroinflammation, and acute‐phase responses. However, evidence supporting their routine use in Long COVID remains limited, and careful patient selection based on inflammatory biomarkers will likely be necessary [39].

Similarly, blockade of interleukin‐1 (IL‐1) using anakinra or canakinumab may attenuate inflammasome‐driven inflammation by suppressing IL‐1β‐mediated immune activation. These therapies may be particularly relevant for patients demonstrating persistent activation of the NLRP3 inflammasome, although robust clinical evidence remains limited. Tumor necrosis factor‐alpha (TNF‐α) inhibitors, including infliximab, adalimumab, and etanercept, have also been proposed for selected inflammatory phenotypes because of their established efficacy in chronic autoimmune diseases. Nevertheless, prospective studies evaluating their efficacy and safety in Long COVID are still lacking [40]. Persistent activation of the alternative and lectin complement pathways has emerged as a promising therapeutic target in Long COVID. Complement activation contributes to endothelial injury, platelet activation, thromboinflammation, leukocyte recruitment, and amplification of cytokine production, making it an attractive target for interrupting chronic inflammatory cycles. Several complement inhibitors are currently under investigation. Eculizumab and ravulizumab, monoclonal antibodies targeting complement component C5, inhibit the formation of the membrane attack complex and generation of the potent inflammatory mediator C5a. Other investigational agents targeting C3, factor D, factor B, or components of the lectin pathway may provide broader regulation of complement‐mediated inflammation. Although clinical evidence in Long COVID remains limited, emerging mechanistic data suggest that complement inhibition may be particularly beneficial in patients with persistent endothelial dysfunction, thromboinflammation, and elevated complement activation biomarkers. Biomarker‐guided patient selection will be essential to maximize therapeutic benefit while minimizing unnecessary immunosuppression [41].

Janus kinase inhibitors offer the advantage of simultaneously suppressing multiple cytokine signaling pathways. Agents such as baricitinib, tofacitinib, and ruxolitinib inhibit intracellular signaling downstream of several cytokine receptors, including IL‐6, interferons, and granulocyte‐macrophage colony‐stimulating factor. Their broad anti‐inflammatory activity has demonstrated benefit in hospitalized patients with acute COVID‐19 and provides a strong rationale for investigation in Long COVID characterized by persistent multisystem inflammation. However, prolonged immunosuppression may increase the risk of opportunistic infections and thromboembolic complications, emphasizing the need for careful risk‐benefit assessment [42]. Mesenchymal stem cell (MSC) therapy has attracted considerable interest because of its potent immunomodulatory, anti‐inflammatory, antifibrotic, and regenerative properties. MSCs secrete extracellular vesicles, growth factors, and anti‐inflammatory cytokines that suppress excessive immune activation, promote regulatory T‐cell expansion, facilitate tissue repair, and restore endothelial integrity. Preliminary clinical studies suggest potential benefits for pulmonary recovery and systemic inflammation, although larger randomized trials are required before routine clinical application [43]. Growing evidence supports a role for gut microbiome dysbiosis in sustaining chronic systemic inflammation through disruption of the gut–lung–brain axis. Therapeutic approaches including probiotics, prebiotics, dietary modification, synbiotics, and fecal microbiota transplantation are being investigated for their ability to restore microbial diversity, improve intestinal barrier function, reduce microbial translocation, and modulate systemic immune responses. While early findings are encouraging, standardized treatment protocols and high‐quality clinical trials remain limited.

Persistent viral antigen stimulation may contribute to ongoing immune activation in a subset of patients with Long COVID. Consequently, prolonged antiviral therapy and interventions targeting viral reservoirs have attracted increasing interest. Although antiviral agents have demonstrated efficacy during acute SARS‐CoV‐2 infection, their role in Long COVID remains uncertain and requires validation through prospective clinical trials. Therapeutic vaccination and immune‐enhancing approaches aimed at improving viral clearance are also under investigation but remain experimental [41]. Persistent inflammation promotes tissue remodeling and fibrosis through activation of transforming growth factor‐beta (TGF‐β), fibroblasts, and extracellular matrix deposition. Antifibrotic agents such as pirfenidone and nintedanib have been proposed for patients with persistent pulmonary fibrosis following COVID‐19, although evidence specific to Long COVID remains limited [42]. Because endothelial dysfunction represents a central pathogenic mechanism, therapies that improve vascular integrity may also provide benefit. These include statins, angiotensin‐converting enzyme inhibitors, angiotensin receptor blockers, and agents that enhance nitric oxide bioavailability or reduce oxidative stress. Their anti‐inflammatory and endothelial‐protective properties warrant further investigation in biomarker‐selected patient populations [43].

Persistent neuroinflammation has prompted investigation of therapies targeting central nervous system immune activation. Low‐dose naltrexone has demonstrated preliminary benefits in reducing microglial activation, neuroinflammation, fatigue, and chronic pain in small observational studies. Additional investigational approaches include modulation of autonomic dysfunction, neuroprotective therapies, and interventions aimed at restoring mitochondrial function and cellular bioenergetics. Although promising, these strategies require rigorous clinical evaluation. The marked biological heterogeneity of Long COVID highlights the limitations of empiric treatment approaches. Future management is expected to rely increasingly on precision immunotherapy, whereby therapeutic selection is guided by comprehensive immune phenotyping rather than clinical symptoms alone. Integration of cytokine profiles, complement activation markers, endothelial biomarkers, coagulation parameters, autoantibody signatures, and multi‐omics data may facilitate identification of biologically distinct disease endotypes that respond preferentially to specific targeted therapies [41]. AI and machine learning are likely to play an increasingly important role in integrating multidimensional datasets, predicting treatment response, and supporting individualized therapeutic decision‐making. However, robust external validation, standardized biomarker assays, and prospective biomarker‐stratified clinical trials are required before precision immunotherapy can be implemented in routine clinical practice (Table 2).

**Table 2:** Integrating cytokine profiles, complement activation, immune‐cell alterations, biomarkers, and potential therapeutic targets in Long COVID.

Immune pathway/mediator	Principal cellular source	Immunopathological role in long COVID	Representative biomarkers	Potential therapeutic targets/strategies	Current evidence
Interleukin‐6 (IL‐6)	Monocytes, macrophages, endothelial cells	Sustains systemic inflammation, endothelial activation, acute‐phase response, B‐cell activation, and neuroinflammation	IL‐6, C‐reactive protein (CRP)	Tocilizumab, Sarilumab, JAK inhibitors	Early clinical studies; requires biomarker‐guided validation
Interleukin‐1β (IL‐1β)	Activated macrophages, monocytes (NLRP3 inflammasome)	Inflammasome activation, chronic inflammation, fever, fibrosis, endothelial injury	IL‐1β, Caspase‐1, ASC, NLRP3	Anakinra, Canakinumab, inflammasome inhibitors	Investigational
Tumor necrosis factor‐α (TNF‐α)	Macrophages, T lymphocytes	Chronic inflammation, endothelial dysfunction, oxidative stress, muscle catabolism	TNF‐α	Infliximab, Adalimumab, Etanercept	Limited clinical evidence
Interferon‐γ (IFN‐γ)	CD4^+^ Th1 cells, CD8^+^ T cells, NK cells	Persistent macrophage activation, antiviral signaling, chronic immune activation	IFN‐γ, CXCL9, CXCL10	JAK inhibitors, immune modulation	Investigational
Interleukin‐17 (IL‐17)	Th17 lymphocytes	Autoimmunity, pulmonary inflammation, fibrosis, neutrophil recruitment	IL‐17	Secukinumab, Ixekizumab (theoretical)	Preclinical rationale; clinical studies needed
Chemokines (CXCL9, CXCL10, CCL2, IL‐8)	Monocytes, macrophages, endothelial cells	Leukocyte recruitment, chronic immune‐cell trafficking, neuroinflammation	CXCL9, CXCL10, CCL2, IL‐8	Chemokine receptor antagonists (investigational)	Emerging evidence
Alternative complement pathway	Liver‐derived complement proteins, activated immune cells	Persistent thromboinflammation, endothelial injury, platelet activation, impaired fibrinolysis	Factor Bb, C3a, C5a, soluble C5b‐9	Factor B inhibitors, Factor D inhibitors, C3 inhibitors	Strong mechanistic evidence; clinical evaluation ongoing
Lectin complement pathway	Mannose‐binding lectin, MASP‐1/2	Complement activation by persistent viral proteins, endothelial injury, inflammation	MASP‐2, C4d, C3a	MASP‐2 inhibitors (e.g., narsoplimab), complement inhibitors	Emerging evidence
Terminal complement activation	Complement cascade	Membrane attack complex formation, vascular injury, inflammation	Soluble C5b‐9, C5a	Eculizumab, Ravulizumab, C5 inhibitors	Early clinical investigation
Monocyte/macrophage activation	Classical and non‐classical monocytes, macrophages	Persistent cytokine production, antigen presentation, chronic innate immune activation	Soluble CD14, Soluble CD163	Cytokine blockade, macrophage‐targeted therapies	Observational evidence
T‐cell dysregulation	CD4^+^ and CD8^+^ T lymphocytes	Persistent activation, immune exhaustion, impaired viral clearance	PD‐1, TIM‐3, activated memory T cells	Immune modulation, therapeutic vaccination (investigational)	Growing evidence
B‐cell activation and autoimmunity	B lymphocytes, plasmablasts	Autoantibody production, immune dysregulation	Autoantibodies, plasmablast frequency	B‐cell‐targeted therapies (e.g., rituximab; investigational)	Limited evidence
Natural killer (NK)‐Cell dysfunction	NK cells	Reduced cytotoxicity and impaired elimination of infected cells	NK‐cell count, cytotoxicity assays	Immunomodulatory approaches	Emerging evidence
Endothelial dysfunction	Endothelial cells	Microvascular injury, vascular inflammation, coagulation activation	VCAM‐1, ICAM‐1, von Willebrand factor, Angiopoietin‐2	Endothelial‐protective therapies, statins, ACE inhibitors (investigational)	Moderate evidence
Coagulation and thromboinflammation	Platelets, endothelial cells	Persistent hypercoagulability, impaired fibrinolysis, microvascular thrombosis	d‐dimer, fibrinogen, thrombin‐antithrombin complexes, PAI‐1	Antithrombotic strategies, complement inhibition (selected patients)	Under investigation
Fibrosis and tissue remodeling	Fibroblasts, macrophages	Pulmonary fibrosis and chronic organ remodeling	TGF‐β, MMP‐9, TIMP‐1	Pirfenidone, Nintedanib	Early clinical studies
Microbiome dysbiosis	Gut microbiota	Gut–lung–brain axis disruption, chronic immune activation	Microbial diversity indices, short‐chain fatty acids	Probiotics, prebiotics, synbiotics, fecal microbiota transplantation	Emerging evidence
Mitochondrial dysfunction	Multiple cell types	Reduced cellular bioenergetics, oxidative stress, fatigue	Lactate, metabolomic signatures	Mitochondrial‐targeted therapies (investigational)	Emerging evidence

Abbreviations: ACE, angiotensin‐converting enzyme; ASC, apoptosis‐associated speck‐like protein containing a caspase recruitment domain; C3a, complement component 3a; C5a, complement component 5a; C5b‐9, terminal complement complex; CCL2, C‐C motif chemokine ligand 2; CRP, C‐reactive protein; CXCL9/CXCL10, C‐X‐C motif chemokine ligands 9 and 10; ICAM‐1, intercellular adhesion molecule‐1; IFN‐γ, interferon‐gamma; IL, interleukin; JAK, Janus kinase; MASP, mannose‐binding lectin‐associated serine protease; MMP‐9, matrix metalloproteinase‐9; NK, natural killer; NLRP3, nucleotide‐binding oligomerization domain‐like receptor family pyrin domain containing 3; PAI‐1, plasminogen activator inhibitor‐1; PD‐1, programmed cell death protein‐1; TGF‐β, transforming growth factor‐beta; TIM‐3, T‐cell immunoglobulin and mucin‐domain containing‐3; TNF‐α, tumor necrosis factor‐alpha; VCAM‐1, vascular cell adhesion molecule‐1.

#### 17.1.1 Biomarker‐Guided Precision Medicine

The considerable clinical and biological heterogeneity of Long COVID underscores the need to move beyond symptom‐based management toward biomarker‐guided precision medicine. Current evidence indicates that Long COVID comprises multiple immunological endotypes characterized by varying degrees of cytokine dysregulation, complement activation, endothelial dysfunction, thromboinflammation, autoimmunity, viral antigen persistence, and metabolic abnormalities. Consequently, a “one‐size‐fits‐all” therapeutic approach is unlikely to provide optimal outcomes. Precision medicine aims to identify the dominant pathogenic mechanisms within individual patients and tailor diagnostic evaluation, risk stratification, and treatment accordingly [44, 45]. A central component of precision medicine is comprehensive immune phenotyping. Rather than relying on individual laboratory markers, integrated biomarker panels combining inflammatory cytokines, complement activation products, endothelial injury markers, coagulation indices, immune cell phenotypes, and tissue remodeling biomarkers provide a more accurate representation of disease biology. Elevated concentrations of interleukin (IL)‐6, IL‐1β, tumor necrosis factor‐alpha (TNF‐α), interferon‐gamma (IFN‐γ), IL‐17, and chemokines such as CXCL9 and CXCL10 may identify patients with persistent cytokine‐driven inflammation, whereas increased levels of complement components including C3a, C5a, soluble C5b‐9, and factor Bb may indicate predominant complement‐mediated thromboinflammation. Similarly, biomarkers such as vascular cell adhesion molecule‐1 (VCAM‐1), intercellular adhesion molecule‐1 (ICAM‐1), von Willebrand factor (vWF), angiopoietin‐2, d‐dimer, and fibrinogen may identify patients with significant endothelial dysfunction and microvascular injury [46, 47, 48].

Emerging evidence suggests that combining multi‐omics technologies with conventional clinical biomarkers can further refine patient stratification. Transcriptomic analyses identify persistent activation of inflammatory, interferon, complement, and coagulation pathways, whereas proteomic profiling reveals alterations in immune mediators, extracellular matrix remodeling proteins, and endothelial biomarkers. Metabolomic and lipidomic studies demonstrate abnormalities in mitochondrial energy metabolism, oxidative stress pathways, and inflammatory lipid mediators, while microbiome analyses identify dysbiosis that may contribute to systemic immune activation through the gut–lung–brain axis. Integration of these datasets enables the identification of biologically distinct disease endotypes that may not be apparent through routine laboratory testing [49, 50]. One promising application of precision medicine is biomarker‐guided therapeutic selection. Patients with dominant IL‐6 signaling may preferentially benefit from IL‐6 receptor antagonists, whereas individuals with persistent inflammasome activation could be candidates for IL‐1 inhibition. Likewise, patients demonstrating sustained complement activation and thromboinflammation may represent appropriate candidates for complement‐targeted therapies currently undergoing clinical evaluation. Biomarkers of endothelial injury, fibrosis, or autoimmune activation may similarly guide the selection of vascular‐protective therapies, antifibrotic agents, or immunomodulatory interventions. Although these approaches remain investigational, they provide a rational framework for individualized treatment rather than empirical therapeutic escalation [51, 52]. AI and machine learning are expected to play an increasingly important role in precision medicine for Long COVID. These computational approaches can integrate high‐dimensional clinical, laboratory, imaging, and multi‐omics data to identify complex biomarker signatures associated with disease severity, clinical phenotypes, and therapeutic response. Predictive algorithms may facilitate early identification of individuals at risk for persistent disease, classify patients into biologically meaningful inflammatory endotypes, and support individualized treatment recommendations. Importantly, explainable AI models are increasingly being developed to improve transparency and clinical interpretability, thereby facilitating integration into routine healthcare. Nevertheless, widespread implementation requires rigorous external validation, standardized data collection, mitigation of algorithmic bias, and adherence to ethical and regulatory standards [53, 54].

#### 17.1.2 Challenges and Future Perspectives

Despite significant advances in understanding the immunopathogenesis of Long COVID, important scientific and clinical challenges continue to impede the development of effective diagnostic tools and targeted therapies. The syndrome is increasingly recognized as a biologically heterogeneous condition encompassing multiple immunological and clinical endotypes rather than a single disease entity. This heterogeneity contributes to substantial variability in symptom profiles, inflammatory signatures, disease trajectories, and treatment responses, making standardized clinical management particularly challenging. One of the foremost challenges is the absence of universally accepted diagnostic biomarkers. Although persistent elevations in proinflammatory cytokines complement activation products, endothelial injury markers, coagulation biomarkers, autoantibodies, and multi‐omics signatures have been reported, no individual biomarker has demonstrated sufficient sensitivity or specificity for routine clinical diagnosis or prognostic assessment. Future studies should prioritize the development and validation of composite biomarker panels that integrate inflammatory, vascular, immunological, and metabolic markers to improve diagnostic accuracy and facilitate biologically informed patient stratification [55]. Another major limitation is the lack of standardized definitions and research methodologies. Variations in diagnostic criteria for Long COVID, patient selection, disease severity during acute infection, vaccination status, circulating SARS‐CoV‐2 variants, duration of follow‐up, and laboratory methodologies contribute to inconsistent findings across studies. Harmonization of clinical definitions, standardized immune phenotyping protocols, and validated laboratory assays will be essential to improve reproducibility and enable meaningful comparisons among future investigations.

Although persistent cytokine dysregulation and complement activation have emerged as central pathogenic mechanisms, their temporal relationships with viral persistence, endothelial dysfunction, thromboinflammation, mitochondrial impairment, autoimmunity, microbiome dysbiosis, and tissue remodeling remain incompletely understood. Future longitudinal studies incorporating serial immune profiling from acute infection through long‐term recovery are needed to define the sequence of immunopathological events and identify critical therapeutic windows. In addition, further investigation is required to determine whether distinct immunological endotypes correspond to specific clinical phenotypes and therapeutic responses. Therapeutically, evidence supporting immunomodulatory interventions remains limited. Most cytokine‐targeted biologics complement inhibitors; primarily mechanistic studies, observational cohorts, or extrapolation from acute COVID‐19 and other immune‐mediated diseases support Janus kinase inhibitors, mesenchymal stem cell therapies, microbiome‐directed interventions, and antifibrotic agents. Large multicenter randomized controlled trials incorporating biomarker‐guided patient selection are urgently needed to establish efficacy, optimize treatment timing, evaluate combination therapeutic strategies, and define long‐term safety profiles. Such studies should also include diverse populations to improve generalizability and reduce healthcare disparities [49].

Rapid advances in multi‐omics technologies—including genomics, transcriptomics, single‐cell sequencing, proteomics, metabolomics, lipidomics, spatial transcriptomics, and microbiome profiling—offer unprecedented opportunities to characterize the molecular heterogeneity of Long COVID. Integration of these technologies with conventional clinical and laboratory data is expected to facilitate identification of robust biomarker signatures, novel therapeutic targets, and biologically distinct disease endotypes. However, widespread implementation remains constrained by high costs, limited accessibility, lack of standardized analytical pipelines, and insufficient external validation. AI and machine learning are likely to become integral components of future Long COVID research and clinical practice. AI‐assisted integration of multidimensional datasets has the potential to improve disease classification, predict long‐term outcomes, identify patients at greatest risk of persistent disability, and support biomarker‐guided therapeutic decision‐making. Nevertheless, successful clinical implementation will require transparent and explainable algorithms, rigorous external validation across geographically and ethnically diverse populations, standardized data acquisition, and careful attention to algorithmic bias, privacy, data governance, and regulatory oversight [47]. Future research should also emphasize international collaboration through large prospective multicenter cohorts with standardized protocols for biospecimen collection, immune profiling, imaging, and long‐term clinical follow‐up. Such collaborative efforts will facilitate validation of candidate biomarkers, improve understanding of disease heterogeneity, and accelerate the translation of laboratory discoveries into clinical practice. Equally important is the inclusion of pediatric populations, older adults, immunocompromised individuals, and populations from low‐ and middle‐income countries, which remain underrepresented in many current studies.

### 17.2 Conclusion

Long COVID has emerged as a complex, heterogeneous multisystem disorder in which persistent immune dysregulation extends well beyond the resolution of acute SARS‐CoV‐2 infection. Current evidence indicates that its pathogenesis is not driven solely by sustained cytokine production but rather by a dynamic interplay among cytokine‐mediated inflammation, persistent complement activation, endothelial dysfunction, thromboinflammation, adaptive immune dysregulation, viral antigen persistence, mitochondrial dysfunction, and tissue remodeling. These interconnected mechanisms contribute to the wide spectrum of clinical manifestations, including fatigue, cognitive impairment, cardiopulmonary dysfunction, dysautonomia, and persistent multisystem symptoms. Recent advances in immunology have substantially improved understanding of the molecular basis of Long COVID. Persistent elevations of inflammatory cytokines—including interleukin (IL)‐6, IL‐1β, tumor necrosis factor‐alpha (TNF‐α), interferon‐gamma (IFN‐γ), and IL‐17—together with sustained activation of the alternative and lectin complement pathways, have emerged as key drivers of chronic inflammation, endothelial injury, and microvascular dysfunction. Complementary insights from transcriptomics, proteomics, metabolomics, microbiome research, and single‐cell technologies further demonstrate that Long COVID comprises multiple biological endotypes rather than a single uniform disease process, emphasizing the need for individualized approaches to diagnosis and treatment.

Although numerous biomarkers have been proposed, including inflammatory cytokines, complement activation products, endothelial injury markers, coagulation indices, immune cell phenotypes, and multi‐omics signatures, none has yet achieved sufficient validation for routine clinical use. Similarly, promising immunomodulatory interventions—including cytokine‐targeted biologics, complement inhibitors, Janus kinase inhibitors, mesenchymal stem cell therapies, microbiome‐directed interventions, and antifibrotic agents—remain largely investigational, with robust evidence from biomarker‐stratified randomized controlled trials still lacking. The future management of Long COVID will likely depend on the integration of validated biomarker panels, comprehensive immune phenotyping, multi‐omics technologies, and AI‐assisted data analysis to identify biologically distinct disease endotypes and guide precision immunotherapy. Such an approach has the potential to improve diagnostic accuracy, optimize therapeutic selection, and enhance long‐term patient outcomes. Continued multidisciplinary research, standardized immune profiling, international collaborative studies, and well‐designed prospective clinical trials will be essential to translate emerging mechanistic insights into safe, effective, and personalized management strategies for individuals living with Long COVID.

## Author Contributions

**Emmanuel Ifeanyi Obeagu:** conceptualization, writing – original draft, methodology, validation, visualization, writing – review and editing, supervision.

## Funding

The author has nothing to report.

## Conflicts of Interest

The author declares no conflicts of interest.

## Transparency Statement

The corresponding author, Emmanuel Ifeanyi Obeagu, affirms that this manuscript is an honest, accurate, and transparent account of the study being reported; that no important aspects of the study have been omitted; and that any discrepancies from the study as planned have been explained.

## Data Availability

Data sharing is not applicable to this article as no datasets were generated or analyzed during the current study.
